# The *don’t know* option in progress testing

**DOI:** 10.1007/s10459-015-9604-2

**Published:** 2015-04-26

**Authors:** C. J. Ravesloot, M. F. Van der Schaaf, A. M. M. Muijtjens, C. Haaring, C. L. J. J. Kruitwagen, F. J. A. Beek, J. Bakker, J. P. J. Van Schaik, Th. J. Ten Cate

**Affiliations:** Radiology Department, University Medical Center Utrecht, Heidelberglaan 100, Room E01.132, 3508 GA Utrecht, The Netherlands; Utrecht University, Utrecht, The Netherlands; Maastricht University, Maastricht, The Netherlands; Albert Schweitzer Hospital, Dordrecht, The Netherlands; Radiology Department, University Medical Center Utrecht, Heidelberglaan 100, 3508 GA Utrecht, The Netherlands; Julius Center, University Medical Center Utrecht, Heidelberglaan 100, 3508 GA Utrecht, The Netherlands; Center for Research and Development of Education, University Medical Center Utrecht, Heidelberglaan 100, 3508 GA Utrecht, The Netherlands

**Keywords:** Construct-irrelevant variance, Construct validity, Don’t know option, Formula scoring, Progress testing, Reliability, Risk-taking tendency

## Abstract

Formula scoring (FS) is the use of a don’t know option (DKO) with subtraction of points for wrong answers. Its effect on construct validity and reliability of progress test scores, is subject of discussion. Choosing a DKO may not only be affected by knowledge level, but also by risk taking tendency, and may thus introduce construct-irrelevant variance into the knowledge measurement. On the other hand, FS may result in more reliable test scores. To evaluate the impact of FS on construct validity and reliability of progress test scores, a progress test for radiology residents was divided into two tests of 100 parallel items (A and B). Each test had a FS and a number-right (NR) version, A-FS, B-FS, A-NR, and B-NR. Participants (337) were randomly divided into two groups. One group took test A-FS followed by B-NR, and the second group test B-FS followed by A-NR. Evidence for impaired construct validity was sought in a hierarchical regression analysis by investigating how much of the participants’ FS-score variance was explained by the DKO-score, compared to the contribution of the knowledge level (NR-score), while controlling for Group, Gender, and Training length. Cronbach’s alpha was used to estimate NR and FS-score reliability per year group. NR score was found to explain 27 % of the variance of FS [F(1,332) = 219.2, *p* < 0.0005], DKO-score, and the interaction of DKO and Gender were found to explain 8 % [F(2,330) = 41.5, *p* < 0.0005], and the interaction of DKO and NR 1.6 % [F(1,329) = 16.6, *p* < 0.0005], supporting our hypothesis that FS introduces construct-irrelevant variance into the knowledge measurement. However, NR-scores showed considerably lower reliabilities than FS-scores (mean year-test group Cronbach’s alphas were 0.62 and 0.74, respectively). Decisions about FS with progress tests should be a careful trade-off between systematic and random measurement error.

## Introduction

Progress testing is used to assess knowledge growth (McHarg et al. 2005). Participants take tests about a knowledge domain across the full breadth of a curriculum at regular intervals during the course. First year students are not expected to answer most items correctly, while final year students are. A “don’t know” option (DKO) is frequently added to stimulate participants to recognize their knowledge deficit (McHarg et al. 2005). In tests with DKOs, “formula scoring” is applied, implying that a penalty is assigned for a wrong answer, while a DKO answer is assigned zero points. With true–false tests the penalty for a wrong answer is −1, and the reward of a correct answer is +1. Accordingly, the test score is defined as the number of correct minus the number of incorrect answers, expressed as a percentage of the maximum score (Kurz 1999). Formula scoring (FS) is thought to reduce random error by minimizing guessing. Participants can choose the DKO when they do not know the answer, otherwise they would be forced to guess which would result in random correct and incorrect answers. The internal consistency among test scores therefore is expected to be higher in tests with DKO, and thus the reliability of these test scores likewise (Burton 2004; Diamond and Evans 1973; Mattson 1965).

Studies on progress tests show that participants’ use of the DKO in general declines with experience (Ravesloot et al. 2012). This can be explained by an increasing knowledge level. However, a drawback of FS is that not only knowledge, but possibly also other constructs are reflected in the test scores (Albanese 1988; Bliss 1980; Diamond and Evans 1973; Kurz 1999). It is likely that not all participants have similar tendencies to choose the DKO (Byrnes et al. 1999; Kampmeyer et al. 2014). Participants who easily take risks may expect that their partial knowledge will make the chance to answer a true–false item correctly generally larger than 50 %. They will less readily choose DKOs. More careful participants may be more inclined to choose the DKO when they are not completely confident about the right answer. Thus, a risk-avoiding participant in general will be disadvantaged and obtain a lower test score than a risk-taking participant with similar knowledge (Messick 1995, 1989; Lord 1963; Muijtjens et al. 1997, 1999). This variation in risk-avoidance decreases the construct validity of the test scores (i.e. the knowledge to be measured) by introducing construct-irrelevant variance (Messick 1995). As it is known that men are generally more inclined to guess than women (Byrnes et al. 1999; Kelly and Dennick 2009), it is important to find out whether women are more disadvantaged by this effect than men.

Effects of FS in course exams have extensively been studied and most authors agree that the disadvantage of FS in course exams outweighs its advantage (Albanese 1988; Bliss 1980; Kelly and Dennick 2009; Kurz 1999; Lord 1975; Muijtjens et al. 1999). Muijtjens et al. found that students with low guessing behavior received lower scores with formula than with number-right scoring, but the reliability of the test scores was found to be higher with FS than with number-right scoring (Muijtjens et al. 1999). It remains unclear whether these findings apply to progress tests as well. Referring to Muijtjens et al., Swanson et al. recommended not to use FS in progress testing (Swanson et al. 2010), and, McHarg et al. (2005) referring to the same article, contrarily recommended to use it because of the reliability benefits. After all, in contrast with course exams, participants of progress tests differ in years of training, and junior students should evidently know fewer answers on the same test than seniors. When a DKO is absent, less experienced participants in progress tests clearly need to guess more than participants in course exams (McHarg et al. 2005; Muijtjens et al. 1999; Ravesloot et al. 2012). This logically reduces the reliability of the test scores of progress tests. However, there is no reported empirical evidence confirming this expectation.

The aim of our study was to empirically evaluate the effect of FS on the construct validity and reliability of progress test scores in postgraduate medical education. The research questions were: (1) Does FS in progress testing introduce construct-irrelevant variance into the knowledge measurement? (2) Does FS in progress tests lead to an increase in reliability of test scores? (3) Are female participants of progress tests more inclined to use the DKO? and (4) Are female participants more disadvantaged by FS than male participants? We hypothesized that all these questions would be answered positively.

We conducted our study using the Dutch Radiology Progress Test (DRPT) in its regular administrations to radiology residents (Ravesloot et al. 2012).

## Method

### Study design

A randomized controlled cross-over design was used to investigate the effect of FS on the construct validity and reliability of test scores of the DRPT. The study was conducted during a regularly administered DRPT (November 2010). One half of the test was administered under FS conditions and the other half of the test under number-right conditions (NR).

Ethical approval for the study was obtained from the Ethical Review Board of the Netherlands Association for Medical Education.

### Sample

The entire population of 379 radiology residents in training in the Netherlands was invited to attend the test; 42 residents (11 %) did not take the test due to, e.g., illness or research periods. All other 337 residents were randomly assigned to two groups (Groups 1 and 2), stratified for training length and gender.

### Instrumentation

The DRPT was introduced in 2003 and has a formative function, in the sense that it provides feedback to residents and program directors on the residents’ knowledge growth (Ravesloot et al. 2012), but does not serve as an exam that must be passed before certification. All Dutch radiology residents take progress tests every 6 months as a mandatory part of their 5-year training program. The tests consist of two hundred true/false items with DKO, covering the complete domain of radiology, as defined in a test blueprint distinguishing nine subdomains: abdominal radiology, neuroradiology, thoracic radiology, musculoskeletal radiology, pediatric radiology, head and neck radiology, cardiovascular and breast imaging. Approximately fifteen percent of the items include an image. Items are constructed by a team of experienced radiologists. Two radiologists construct items for one subdomain which are subsequently reviewed by the other team members. The scores are calculated using FS. Reliabilities have been consistently high (Cronbach’s alpha across year groups was 0.87 on average for all nine examinations from 2005 to 2010). See for detailed information on the quality and procedures of the DRPT Ravesloot et al. 2012.

The format, content, item construction and procedures were similar to previous DRPT administrations. For this experiment, the 200 test items were divided into two subsets of 100 items, tests A and B. Items across radiological subdomains and across item writers were evenly allocated to test A and B. The 200 items were constructed following the normal procedure, and subsequently all items per subdomain and item writer were split up into two subsets to compose two ‘parallel’ tests of 100 items. Each test had a FS version (A-FS and B-FS) and a number-right scoring version (A-NR and B-NR).

### Procedure and measurements

Group 1 started with test A-FS and Group 2 started with test B-FS. After they had completed the first test, they proceeded with the second test in number-right format (Group 1 test B-NR, and Group 2 test A-NR). It was necessary to let the groups take different tests in the FS version, because otherwise a difference in score between the two conditions (formula and number-right scoring) could be due to content and difficulty differences of the two tests instead of difference in condition. This cross-over design in fact constitutes a duplication of our experiment.

Both groups started with the FS version of the test to prevent a behavior change caused by the, for participants unusual, number-right scoring test, when taking the FS test. In this way, we avoided a spill-over effect of the intervention.

Scores on the FS test were calculated with FS, and scores on the number-right test were calculated by adding all correct answers (NR). Scores were calculated as a percentage of the maximum score (number of items in the test). Participants were informed about the scoring rules in advance.

### Measurements

Per participant the percentage DKO answers (DKO score), and the number of correct minus incorrect answers expressed as a percentage of the number of items (FS score), were calculated using the test data obtained under FS conditions. The DKO score was considered to be an indicator of the level of risk avoidance of the participant. The percentage correctly answered items calculated from test data obtained under number-right conditions (NR score) was used as an indicator of the knowledge level of the participant. For each participant the training length (TR) was calculated (Ravesloot et al. 2012).

### Data analysis

#### Baseline characteristics of groups and tests

Mean A-FS and B-FS scores and A-NR and B-NR scores were compared (*t* tests), to check for difficulty differences. Effect sizes were calculated using Cohen’s d, where 0.2, 0.5, and 0.8 are considered to indicate small, moderate, and large effects, respectively (Cohen 1988).

#### Construct validity

To study the effect of the DKO on construct validity of FS test scores (research question 1), we investigated risk taking tendency, which is influenced by gender and personality, as a major source of construct irrelevant variance in progress test scores, that are obtained under FS conditions. The DKO score is assumed to reflect the tendency to avoid risk, i.e. a higher DKO score indicates a participant with a higher level of risk avoidance. However, the DKO score is also influenced by experience and knowledge. Compared to participants with lower levels of knowledge or experience, but with similar risk taking tendency, participants with a high level of knowledge/experience will have a lower DKO score. By studying the effect of the DKO score on the test score obtained under FS conditions (FS score), while controlling for knowledge level (NR score) and experience (TR), we obtain an indication of the net effect of risk avoidance per se on the FS score. If there is such effect, participants with a more risk taking tendency most likely would achieve higher FS scores than participants with less risk taking tendency.

To estimate this hypothesized effect, we conducted a sequential (hierarchical) multiple regression analysis after an assumption check on outliers, normality and multicollinearity. In the analysis we investigated how much of the variance of FS is explained by NR, and how much in addition is explained by DKO. The first being the desired influence of a person’s knowledge level, the latter representing the undesired influence of a person’s tendency for risk avoidance, which in the context of knowledge measurement is considered a construct-irrelevant contribution. The sequential regression analysis is performed in a stepwise manner, extending the model in each step and using the corresponding change in explained variance (R^2^ change) as an indicator of the explanatory power of the added independent variable, respectively, the square root of R^2^ change as an indicator of the importance of this contribution (Keith 2006). In addition to NR and DKO as the primary independent variables of interest, there are a number of background variables we need to control for in the analysis: Gender, TR (training length), and Group. Group was added to control for difficulty differences between tests A and B. Furthermore, in order to test whether the effect of risk avoidance on FS was moderated by knowledge level, an interaction term (NR × DKO) was added to the model. For convenience of interpretation, all continuous independent variables (DKO score, NR score and TR) were centered by subtracting the corresponding mean value. For scaling purposes interaction NR × DKO was defined as (NR score × DKO score)/(SD of NR score). Thus the regression coefficient of the interaction indicates the change of the effect of DKO score on FS score per one SD-step change in the knowledge level (NR score) of a participant.

In order to investigate whether the influence of the independent variables on the variance of FS is different for female versus male (research question 4), interactions of Gender with all other independent variables (IV) were obtained by calculating product variables Gender × IV, and including these as additional independent variables in the sequential regression analysis.

Generally, in multiple regression the standard regression coefficient can be used as an indicator of effect size, and according to Cohen’s classification values 0.1, 0.3, and 0.5 indicate small, moderate and large effects, respectively (Cohen 1988). These classification values can also be used to interpret the substantiality of the square root of the R^2^ change in the analyses.

#### Reliability

To answer research question 2, reliabilities were estimated with Cronbach’s alpha for tests A-FS, B-FS, A-NR, and B-NR for each training year. The analysis focused on reliability differences per year group because it is the ability of the test to distinguish between low and high performers within a year group that is of interest here. Differences in reliability between formula and number-right scoring tests were tested using the K-sample significance test for independent alpha coefficients (Hakstian and Whalen 1976).

#### Gender and tendency to use DKO

The effect of gender on DKO score (research question 3) was evaluated by regression analysis. After an assumption check on outliers, normality and multicollinearity, simultaneous multiple regression analysis was conducted using the DKO score as the dependent variable, Gender, as the independent variable of interest, and NR score, TR, and Group as controlling variables.

Analyses were performed using IBM SPSS Statistics 20 (IBM Corp. 2011). Results were considered statistically significant if *p* < 0.05.

## Results

### Baseline characteristics groups and tests

All 337 residents participated in the study (168 in Group 1 and 169 in Group 2). Mean scores and group characteristics are shown in Table 1. Three items in test A and two items in test B with a negative corrected item-total correlation in both conditions (FS and NR) were eliminated from the dataset. Mean NR scores for group 1 (B-NR) were significantly higher [*t*(355) = 2.2, *p* < 0.05] than for group 2 (A-NR), Cohen’ *d* = 0.24. Mean FS scores for the two groups (A-FS and B-FS) were not significantly different. Test B appeared to be less difficult despite the even allocation of test items to both tests. However, the effect size of the difference, was small.

**Table 1:** Group characteristics and test results

Variables	Group 1	Group 2
	Tests A-FS, B-NR	Tests B-FS, A-NR
Number of participants (n)	168	169
Gender (male:female)	95:73	94:75
TR, training length in years, Mean (SD)	2.4 (1.4)	2.3 (1.4)
FS score, percentage correct minus incorrect under FS conditions, Mean (SD)	A-FS 31.5 (17.4)	B-FS 34.3 (16.3)
NR score, percentage correct under NR conditions, Mean (SD)	B-NR 67.5 (9.6)	A-NR 65.4 (7.9)
DKO score, percentage don’t know under FS conditions, Mean (SD)	26.4 (19.2)	29.8 (19.8)

### Construct validity

DKO, NR, TR, gender, group, the interaction NR × DKO, and interactions of each of the independent variables with Gender were entered as predictors in a sequential multiple regression model to assess their contribution to the explanation of the variance of FS score. Assumptions for multiple regression analysis were found not to be violated. Exclusion of outliers (eight cases) resulted in negligible changes in the main effects. Only the coefficient of the interaction DKO × NR changed from −0.11 to −0.09, but still significant, and not resulting in a substantial change of the conclusions. Therefore we decided to report the regression results for the complete dataset. None of the interactions with Gender was found to have a significant contribution to FS variance except Gender × DKO score, so this was the only interaction with Gender included in the final regression analysis (see Table 2).

**Table 2:** Results of the sequential (hierarchical) multiple regression analysis of dependent variable formula scoring (FS) score with independent variables Gender, training length TR), group, number right (NR) score, don’t know option (DKO) score, and the interactions of Gender and DKO score (Gender × DKO), and of NR score and DKO score (NR score × DKO score)

Model	Independent variable	Regression coefficient			Standardized regression coefficient β	R^2^ change ∆R^2^	Importance $\sqrt {\varDelta R^{2} }$	R^2^
		b	*p*	SE				
1						0.327	0.57	0.33
	Intercept	32.13	0.000	1.26				
	Group	2.91	0.056	1.52	0.09			
	Training-length (years)	6.61	0.000	0.53	0.57			
	Gender (0:male; 1:female)	−1.54	0.315	1.53	−0.05			
2						0.268	0.52	0.60
	Intercept	31.09	0.000	0.98				
	Group	5.31	0.000	1.19	0.16			
	Training-length	2.56	0.000	0.49	0.22			
	Gender	−1.90	0.110	1.19	−0.06			
	Number right score	1.20	0.000	0.08	0.63			
3						0.081	0.28	0.68
	Intercept	30.27	0.000	0.89				
	Group	5.93	0.000	1.07	0.18			
	Training-length	0.40	0.434	0.51	0.03			
	Gender	−0.94	0.380	1.07	−0.03			
	Number right score	0.99	0.000	0.08	0.52			
	Don’t know option score	−0.39	0.000	0.04	−0.45			
	Gender × DKO score	0.14	0.014	0.06	0.11			
4						0.016	0.13	0.69
	Intercept	28.99	0.000	0.92				
	Group	6.37	0.000	1.05	0.19			
	Training-length	0.29	0.560	0.50	0.03			
	Gender	−1.26	0.232	1.05	−0.04			
	Number right score	0.96	0.000	0.08	0.50			
	Don’t know option score	−0.43	0.000	0.04	−0.50			
	Gender × DKO score	0.10	0.078	0.06	0.08			
	NR score × DKO score	−0.11	0.000	0.03	−0.14			

Number right score and don’t know option score are expressed as percentages of the maximum attainable score

Continuous independent variables training-length, number right score and don’t know option score are centered on their mean value

For scaling purposes interaction NR score × DKO score is defined as (NR score × DKO score)/(standard deviation of NR score)

In the first step (Model 1) of the final sequential analysis the background variables Group, TR, and Gender were entered, and were found to explain 33 % of the variance of FS [R^2^ = 0.33; F(3,333) = 54.0, *p* < 0.0005], where almost all of the explained variance is due to Training length. So a considerable part of the variance in FS is explained by differences in Training length of the participants (β = 0.57), where more training-years result in a higher score (*t* test, *p* < 0.0005, b = 6.61). When NR was added (Model 2) R square increased to 60 %, a substantial increase of the explained variance of FS with 27 % [F(1,332) = 219.2, *p* < 0.0005]. Subsequently, adding DKO and Gender × DKO (Model 3) results in a further increase of 8 % of R^2^ [F(2,330) = 41.5, *p* < 0.0005], and finally adding the interaction NR × DKO (Model 4) increases R square with another 1.6 % [F(1,329) = 16.6, *p* < 0.0005], resulting in a final R^2^ of 69 %.

To compare the impact of the contributions of NR, DKO&Gender × DKO, and NR × DKO it is recommended (Keith 2006) to consider the square root of R^2^ change (column ‘Importance’ in Table 2), indicating that NR is an important explaining variable for FS, the contribution of DKO&Gender × DKO being substantial, but only about half as important as NR’s, and finally, the contribution of NR × DKO being only a quarter as important as NR’s.

The total effect of NR (Model 2) was highly significant (*t* test, *p* < 0.0005) with a standard regression coefficient (β) of 0.63, indicating a large effect. The results for Model 3 show that controlled for NR and the background variables, there is a highly significant (*t* test, *p* < 0.0005) influence of risk avoidance tendency on FS (b = −0.39; β = −0.45) lowering FS, but this disturbing influence is found to be smaller for female participants: for Gender × DKO the effect is b = 0.14, so the net effect for women is b = −0.39 + 0.14 = −0.25, while for men it is −0.39.

Finally, Model 4 shows that the effect of NR × DKO is highly significant (*t* test, *p* < 0.0005), with b = −0.11 indicating that the disturbing influence of DKO is larger for the better students (having a higher level of knowledge), though beta = −0.14 shows that the effect is small. In Model 4, in addition to the highly significant effects of NR and DKO (*t* test, *p* < 0.0005, β = 0.50, and −0.50, respectively), also Group showed a significant effect on FS (*t* test, *p* < 0.0005, b = 6.37) indicating that, the FS scores for Group 2 (test B) were higher compared to Group 1 (test A), and, hence, also when controlling for Gender, TR, NR, DKO, Gender × DKO, and NR × DKO, test A was found to be more difficult than test B.

Note: In the final model the contributions of NR and DKO still are considerable, but of equal importance, beta being equal to 0.50, and −0.50, respectively, while the findings in Models 2, and 3 showed a contribution of NR which was almost twice as large as DKO’s contribution. The reason for this apparent contradiction is as follows. In Model 2 the contribution of NR corresponds to the total effect of NR on FS, hence, the direct effect of NR on FS, as well as the indirect effect of NR on FS, via DKO as a mediating variable. In Model 4, however, for NR only the direct effect is estimated, because DKO is included and thus is controlled for. As we were interested in the disturbing influence of DKO we should compare the additional effect of DKO with the total effect of NR, as we did in the above analysis.

### Reliability

In Table 3 reliabilities (Cronbach’s alpha) are shown for each year-test group (varying training length). In general the reliability for NR scores was significantly and substantially lower than for FS scores for both tests A and B when using the K-sample significance test for independent alpha coefficients (Hakstian and Whalen 1976). The only exceptions were (1) test A in year 3, which showed a difference in the same direction, but not statistically significant, and (2) test B in year 5, which showed a higher reliability for the NR score than for the FS score. For nine out of 10 year-test groups the reliability for the FS score was found to be higher than for the NR score, a result when applying a Sign Test appeared statistically significant (one sided *p* = 0.011). For these 9 year-test groups, in addition to the 100 items in the current test, between 33 and 230 extra items would be needed (according to the Spearman Brown formula) for the test taken under NR conditions to achieve the same level of reliability as obtained with the test taken under FS conditions.

**Table 3:** Reliability (Cronbach’s alpha) obtained with tests A and B under formula scoring conditions (tests A-FS and B-FS) and number-right conditions (tests A-NR and B-NR) in each of the five postgraduate year groups, with the residents divided into experimental Groups 1, and 2

Postgraduate year group	Test conditions			
	Formula scoring	Number-right	Formula scoring	Number-right
	Group 1^a^	Group 2	Group 2	Group 1
	Test A-FS^b^	Test A-NR	Test B-FS	Test B-NR
1	0.70*	0.55*	0.81**	0.56**
2	0.77**	0.58**	0.76*	0.65*
3	0.69	0.63	0.81*	0.72*
4	0.78**	0.61**	0.70*	0.58*
5	0.75**	0.56**	0.59**	0.79**

Significance of difference in reliability formula scoring versus number-right: * *p* < 0.05; ** *p* < 0.001

^a^Number of residents in experimental Groups 1, and 2: 168, and 169, respectively

^b^Number of items in tests A and B: 97, and 98, respectively

### Gender and DKO option use

After assumption checks (multicollinearity, outliers and normality) revealed no violations simultaneous multiple regression analysis was performed to estimate the effect of gender on DKO score, while controlling for Group, TR and NR (Table 4). Gender was found to significantly contribute to the variance of DKO option use (*t* test, *p* = 0.045, b = 3.05), showing a higher use for female versus male participants, although the effect is small (β = 0.08).

**Table 4:** Results of the simultaneous multiple regression analysis of dependent variable don’t know option (DKO) score with independent variables group, training length (TR), number right (NR) score, and Gender

Independent variable	Regression coefficient			Standardized regression coefficient	R^2^
	b	*p*	SE	β	
					0.51
Intercept	25.84	0.000	1.25		
Group	1.88	0.216	1.51	0.05	
Training-length (years)	−6.62	0.000	0.63	−0.49	
Number right score	−0.68	0.000	0.10	−0.31	
Gender (0:male; 1:female)	3.05	0.045	1.51	0.08	

Number right score and don’t know option score are expressed as percentages of the maximum attainable score

Continuous independent variables training-length, and number right score are centered on their mean value

## Discussion

### Construct validity

Our results show that for participants with the same training length, the larger part (27 %) of the FS score variance is explained by the variation in knowledge level, as represented by the number right (NR) score, indicating that FS score indeed measures to a large extent a participant’s knowledge level. However, using FS, the knowledge measurement is disturbed by differences in the tendency to use the don’t know option (DKO) among participants of the same knowledge level, i.e. showing varying levels of risk avoidance. The contribution of the DKO score accounts for another 8 % of the FS score variance, and in terms of importance as explaining variable, the contribution of the disturbance is more than half as important as the knowledge based contribution, and, hence, is considerable. So, indeed as anticipated, the use of FS does weaken the construct validity of a test score intended to measure a participant’s knowledge level, because of the disturbing effect of, for example, risk avoidance variation. For female versus male participants this disturbing effect was shown to be smaller: a female participant having the same knowledge and the same tendency to use the don’t know option as a male participant, in general will obtain a higher FS score. So, women compared to men are better in optimally using a given number of don’t know options in a test, i.e. are better in estimating their chances to answer correctly with questions they only have partial knowledge of. On the other hand: compared to men, women were shown to have a higher tendency to use the don’t know option, a finding which is consistent with the sparse research done in the past (Byrnes et al. 1999; Kelly and Dennick 2009). So the question is: what is the net effect for the FS score of women? Will it be lower or higher compared to men of similar training length and knowledge (NR)? The answer can be found in the Model 2 analysis in Table 2, and the outcome is: there is no significant effect of gender. So, the better use of the don’t know option by women is apparently compensated by their tendency to more frequently use the DKO (higher level of risk avoidance), and the net result is no advantage/disadvantage for women versus men with FS.

Participants with higher knowledge levels were more disadvantaged by using the DKO in FS tests compared to participants with a lower knowledge level. A possible explanation is that the partial knowledge of the well-performing residents is higher, and this source is not exploited, when the resident is too reluctant to answer with FS. Consequently, the chance to guess right would be higher for participants with more knowledge, so they are expected to benefit more from answering an item without using the DKO than participants with less knowledge (Muijtjens et al. 1999).

Our study indicates that the DKO weakens the construct validity of progress tests, because it introduces construct-irrelevant variance in the test results. However, this finding should be interpreted with some caution. First, we could not measure individual risk taking tendency directly. Instead, we took the use of the DKO at similar levels of knowledge and experience as a proxy. Theoretically, participants with similar DKO use can have a different risk taking tendency, explained by differences in knowledge level and/or experience. However, in our analysis this confounding was prevented by including knowledge level and experience level as independent variables in the regression model. However, it would be useful to evaluate the effect of risk taking on formula scores using questionnaires measuring individual risk taking in progress testing in future research.

Second, with FS participants are also tested on their self-reflective ability, which is not assessed in number-right scoring. In other words, what does “construct-irrelevant” variance mean? If it is the purpose of the test to assess self-reflective ability of the participants than the DKO and the effect on the test scores implies “construct-relevant” variance. This is mentioned in other studies as an advantage of FS in medical education, as doctors are not expected to merely guess in clinical practice but should be able to evaluate whether they do or do not have sufficient knowledge to make an informed guess (Muijtjens et al. 1999). The progress test in the current study includes true/false items. However, the conclusions about the effect of DKO on progress test validity hold for multiple-choice items progress tests with more options as well. When increasing the number of alternatives the penalty for incorrect answers will be adapted, so that random guessing will lead to an average score of zero points. For example, in a four-options MCQ test the penalty for incorrect answers is 0.33 points. With random guessing of four questions this will lead to an expected score of (3 × −0.33) − 1 = 0 points.

### Reliability

We hypothesized that the reliability of test scores would decrease when number-right scoring is used instead of FS. As we expected, the reliability was indeed lower for the number-right scores than for the formula scores as shown by the Cronbach’s alpha decrease for nine out of ten group comparisons. However, the severity of the reliability decrease might differ in distinct populations. Theoretically, the larger the number of items that cannot be answered, the larger a decrease in reliability is expected to occur when removing the DKO option from the test. The number of items juniors are able to answer differs between settings and domains. In our study, first year residents use the DKO on average with 60 % of the test items (Ravesloot et al. 2012). Probably, residents are never completely ‘blank’ at residency enrolment, and knowledge obtained in undergraduate medical training can be used by juniors to answer postgraduate test items. In undergraduate medical progress testing DKO scores among juniors can be considerably larger. Blake et al. (1996) reported that their first year students only answered approximately 20 % of the items, which is equivalent to a DKO score of 80 %. In this case the reliability decrease is expected to be larger when the DKO is removed.

Effects of an additional DKO on the reliability of test scores will probably not differ between true/false and multiple-alternatives item test scores, because the decrease in random error will be comparable.

### Formula or number-right scoring?

Choosing between formula and number-right scoring means choosing between bias and random measurement error in progress test results. Important arguments to consider in this dilemma are:

1. What is the purpose of the test? Is it aimed at merely estimating the knowledge level, or is it also aimed at finding out how participants use their knowledge, including self-assessment of their abilities? Especially, in undergraduate medical education, where first years are not able to answer the vast majority of test items, the DKO might be of more value to learn students that it is important to acknowledge your deficits. Number-right scoring might be more suitable for the first purpose, and FS for the second;
2. What is the amount of DKO use, i.e. random guesses in NR, among different training years? High DKO use might result in a preference for FS. The drawback of a low reliability per test might be acceptable if summative conclusions are based on a combination of scores of several tests. The decision for FS or NR then depends on the level of reliability achieved with the combined test scores.
3. Is it possible to sufficiently overcome the reliability decrease in NR, e.g. by adding test items or by improving discriminative power of individual items? If this is the case, it might favor NR.

## Conclusions

Our results add to the evidence that using a DKO weakens the construct validity in progress testing. The effect of this disadvantage is considerable, and disproportionally affect participants with more knowledge. Men and women were evenly afflicted by the effect: compared to men, women were shown to more frequently use the don’t know option, but they were able to compensate the corresponding averse effect by a better use of the don’t know option. As anticipated, the reliability of the DRPT decreases when changing from formula to number-right scoring. Decisions about DKO use in progress tests should be a careful trade-off between systematic (bias) and random measurement error.
